# An Optimized NGS Workflow Defines Genetically Based Prognostic Categories for Patients with Uveal Melanoma

**DOI:** 10.3390/biom15010146

**Published:** 2025-01-18

**Authors:** Michele Massimino, Elena Tirrò, Stefania Stella, Cristina Tomarchio, Sebastiano Di Bella, Silvia Rita Vitale, Chiara Conti, Marialuisa Puglisi, Rosa Maria Di Crescenzo, Silvia Varricchio, Francesco Merolla, Giuseppe Broggi, Federica Martorana, Alice Turdo, Miriam Gaggianesi, Livia Manzella, Andrea Russo, Giorgio Stassi, Rosario Caltabiano, Stefania Staibano, Paolo Vigneri

**Affiliations:** 1 Department of General Surgery and Medical-Surgical Specialties, University of Catania, 95123 Catania, Italy; andrea.russo@unict.it; 2 Center of Experimental Oncology and Hematology, A.O.U. Policlinico “G. Rodolico-S. Marco”, 95123 Catania, Italy; ele_tir@yahoo.it (E.T.); or stefania.stella@unict.it (S.S.); cristina.tomarchio@hotmail.it (C.T.); silviarita.vitale@gmail.com (S.R.V.); manzella@unict.it (L.M.); 3 Department of Clinical and Experimental Medicine, University of Catania, 95123 Catania, Italy; fede.marto.fm@gmail.com (F.M.); vigneripaolo@gmail.com (P.V.); 4 Department of Precision Medicine in Medical, Surgical and Critical Care (Me.Pre.C.C.), University of Palermo, 90127 Palermo, Italy; sebastiano.dibella@unipa.it (S.D.B.); miriam.gaggianesi@unipa.it (M.G.); giorgio.stassi@unipa.it (G.S.); 5 Department of Human Pathology “G. Barresi”, University of Messina, 98125 Messina, Italy; conchiara9@gmail.com (C.C.); marialuisapuglisi94@gmail.com (M.P.); 6 Pathology Unit, Department of Advanced Biomedical Sciences, University of Naples Federico II, 80131 Naples, Italy; rosamariadicrescenzo@gmail.com (R.M.D.C.); silvia.varricchio@unina.it (S.V.); stefania.staibano@unina.it (S.S.); 7 Department of Medicine and Health Sciences “V. Tiberio”, University of Molise, 86100 Campobasso, Italy; francesco.merolla@unimol.it; 8 Department of Medical and Surgical Sciences and Advanced Technologies “G.F. Ingrassia”, Anatomic Pathology, University of Catania, 95123 Catania, Italy; giuseppe.broggi@gmail.com (G.B.); rocaltab@unict.it (R.C.); 9 Department of Health Promotion, Mother and Child Care, Internal Medicine and Medical Specialties (PROMISE), University of Palermo, 90127 Palermo, Italy; alice.turdo@unipa.it; 10 Division of Oncology, Humanitas Istituto Clinico Catanese, Misterbianco, 95045 Catania, Italy

**Keywords:** molecular profiling, TCGA, uveal melanoma, NGS

## Abstract

Background: Despite advances in uveal melanoma (UM) diagnosis and treatment, about 50% of patients develop distant metastases, thereby displaying poor overall survival. Molecular profiling has identified several genetic alterations that can stratify patients with UM into different risk categories. However, these genetic alterations are currently dispersed over multiple studies and several methodologies, emphasizing the need for a defined workflow that will allow standardized and reproducible molecular analyses. Methods: Following the findings published by “The Cancer Genome Atlas–UM” (TCGA-UM) study, we developed an NGS-based gene panel (called the UMpanel) that classifies mutation sets in four categories: initiating alterations (*CYSLTR2*, *GNA11*, *GNAQ* and *PLCB4*), prognostic alterations (*BAP1*, *EIF1AX*, *SF3B1* and *SRSF2*), emergent biomarkers (*CDKN2A*, *CENPE*, *FOXO1*, *HIF1A*, *RPL5* and *TP53*) and chromosomal abnormalities (imbalances in chromosomes 1, 3 and 8). Results: Employing commercial gene panels, reference mutated DNAs and Sanger sequencing, we performed a comparative analysis and found that our methodological approach successfully predicted survival with great specificity and sensitivity compared to the TCGA-UM cohort that was used as a validation group. Conclusions: Our results demonstrate that a reproducible NGS-based workflow translates into a reliable tool for the clinical stratification of patients with UM.

## 1. Introduction

Uveal melanoma (UM) is the most common ocular cancer arising from the malignant transformation of melanocytes localized in the uveal tract. Although UM is a rare tumor, its high dissemination properties make it a deadly disease [1,2]. UM incidence varies according to sex, race and country. Males have a 30% higher probability of developing the disease compared to females. The incidence in Europe is higher in northern regions (i.e., Denmark: 8/1,000,000) than in southern countries (i.e., Spain: 2/1,000,000), with a lower number of cases in the African American and Asian populations (0.3/1,000,000) [2].

Aside from Tebentafusp-tebn, an FDA (Food and Drug Administration)-approved immunotherapeutic drug effective in HLA-A*02:01-positive patients [3,4,5], there are currently no pharmacological treatments that can increase the survival rate of individuals diagnosed with metastatic UM. Hence, the early identification of prognostic biomarkers may greatly impact disease monitoring and management. Available genomic studies have reported specific genetic alterations that cluster patients with UM into prognostically distinct categories [6,7]. Specifically, a study performed by The Cancer Genome Atlas (TCGA) project on 80 patients with UM (TCGA-UM) showed that the mutation status of specific genes and the imbalance of defined chromosomes are associated with overall survival rates. Indeed, this report identified two groups of mutated genes: initiating genes (*CYSLTR2*, *GNA11*, *GNAQ* and *PLCB4*) and genes with prognostic impact (*BAP1*, *EIF1AX*, *SF3B1* and *SRSF2*). In terms of chromosomal abnormalities, the monosomy of chromosome 3 was associated with the highest metastatic potential, and its association with chromosome 8 alterations further increased the reliability of its OS estimation power [8]. Alterations on chromosome 1 have also been investigated and may represent an additional prognostic biomarker [9,10].

Overall, these observations highlight the need for a detailed workflow that identifies prognostic parameters that may improve the clinical management of patients with UM. Here, we describe a custom DNA-based NGS panel that identifies genetic alterations that allow the precise molecular characterization of patients with UM starting from surgical enucleation.

## 2. Materials and Methods

### 2.1. Sample Collection

Formalin-fixed, paraffin-embedded (FFPE) tumor tissues were obtained from 44 Italian patients with UM retrospectively recruited by the University of Naples “Federico II”, selected according to the availability of archival and clinical data. Each tissue specimen, derived from ocular enucleation, was used for DNA isolation and molecular profiling. 

### 2.2. DNA Extraction and Quantification

For each specimen, 5-micron-thick slides were generated from FFPE samples, and tumor content was determined with hematoxylin and eosin staining by a dedicated pathologist. All samples presented 85–95% tumor cellularity. Genomic DNA (gDNA) was isolated using the automatic QIAsymphony platform employing a DNA mini kit according to the manufacturer’s protocol (both from Qiagen, Hilden, Germania). Isolated gDNA was then quantified using the Qubit 4.0 fluorometer with a dsDNA HS Assay kit (both from ThermoFisher, Waltham, MA, USA). The quality and purity of isolated gDNA was measured by capillary electrophoresis using the Qiaxcell instrument with the DNA High-Sensitivity kit (All from Qiagen).

### 2.3. NGS Cancer Panel Design (UMpanel)

An NGS cancer panel, named the UMpanel, was generated using the Ion AmpliSeq Designer tool (https://www.ampliseq.com/login/login.action, accessed on 21 July 2022) (ThermoFisher) on chromosome coordinates based on the hg19 (GRCh37) reference genome. The panel encompasses 418 amplicons in 2 primer pools recognizing 15 genes. *BAP1*, *CDKN2A*, *CENPE*, *FOXO1*, *HIF1A*, *RPL5*, *SRSF2* and *TP53* are targeted for their entire coding region (CDS), while for *BRAF*, *CYSLTR2*, *EIF1AX*, *GNA11*, *GNAQ*, *PLCB4* and *SF3B1*, we selected the more relevant exon alterations (Supplementary Table S1). In addition, 55 primer pairs were chosen for the analysis of specific single-nucleotide polymorphisms (SNPs) to detect imbalances in chromosomes 1, 3 and 8 as previously reported [9] (Supplementary Tables S2 and S3).

### 2.4. NGS Library Preparation

Ten nanograms per primer pool of gDNA was used for multiplex PCR employing the Ion Ampliseq Library kit 2.0 for both the UMpanel and the Ion AmpliSeq Cancer HotSpot Panel v2 (CHPv2), followed by barcoding with the IonExpress adapter kit (all from ThermoFisher). Each library was then quantified by qPCR with the Ion Library TaqMan Quantification kit (ThermoFisher) employing the ABI 7500 Real-Time PCR System (Applied Biosystem, Waltham, MA, USA). We used the automatic Ion Chef platform for template preparation with each chip employed for NGS library sequencing using the Ion GeneStudio S5 plus System (all from ThermoFisher).

### 2.5. NGS Data Analysis

The Ion Torrent Suite v.5.12.3 was used to control the chip loading density, median read length and percentage of both mapped reads and uniformity. Sequenced raw data were aligned to the hg19 (GRCh37) reference genome to obtain the relative BAM (Binary Alignment Map) file, which was exported to Ion Reporter Software v.5.18 for SNV (Single-Nucleotide Variation), Ins/Del (Insertions/Deletions) and SNP (Single-Nucleotide Polymorphism) annotation. For variant calling, we used a filter chain, applying a VAF (variant allele frequency) cut-off of 10%, read depth > 100, Phred quality score > 40 and *p* < 0.01 to eliminate false positives. All nucleotide variants were filtered according to the interpretation reported in the ClinVar, Cancer Mutation Census and classified as Pathogenic, VUS (Variant with Uncertain Significance), CIP (Conflicting Interpretation of Pathogenicity) or novel, while all variants categorized as benign or common SNPs in ClinVar and in the UCUS genome browser were excluded. Nucleotide variations causing synonymous variants in the protein sequence were also removed. Tile plots were generated using GraphPad Prism v.8.0.

### 2.6. Performance Evaluation of the UMpanel

To evaluate the performance of our workflow, we compared the VAFs obtained with the UMpanel with those observed using the commercially available CHPv2 or the OncoSpan DNA reference (Horizon Discovery, Waterbeach, UK). Concordance was evaluated by calculating sensitivity, specificity and Cohen’s κ. For Cohen’s values, the agreement was considered weak with a κ < 0.4, moderate when κ was between 0.4 and 0.8 and strong for κ values > 0.8. Moreover, we used PCR validation followed by Sanger sequencing for eight common driver genes (*BAP1*, *CYSLTR2*, *EIF1AX*, *GNA11*, *GNAQ*, *PLCB4*, *SF3B1* and *SRSF2*) and for a newly identified *BRAF* mutation. For the detection of chromosome imbalances, SNP analysis was validated employing the standard gDNA NA12878. This sample is a pilot genome derived from the international HapMap (haplotype map) project focused on the analysis of variations in human DNA sequences. NA12878 is also certified by the Coriell Institute as devoid of chromosomal aberrations.

### 2.7. Sanger Sequencing

HotSpot mutations for the *BAP1*, *BRAF*, *CYSLTR2*, *EIF1AX* (exons 1 and 2), *GNA11* (exons 4 and 5), *GNAQ*, *PLCB4*, *SF3B1* and *SRSF2* genes were analyzed by the Sanger sequencing of PCR fragments obtained using the primers reported in Supplementary Table S4. All amplicons were generated using the Platinum™ PCR SuperMix High Fidelity (ThermoFisher) according to the manufacturer’s instructions, with annealing performed 5 °C below the melting temperature. PCR products were separated on 1.8% agarose gels and then purified employing the QIAEX II Gel extraction kit (Qiagen).

### 2.8. Statistical Analysis and Overall Survival Estimation

To analyze data concordance, linear regression was applied using Prism Software v.8.0 (Irvine, CA, USA), which was also employed for the identification of outliers [11]. To compare our findings with those of the TCGA analysis, we extracted patients’ data from the TCGA-UM cohort (sample: *n* = 80), focusing on the mutational status of *BAP1* and on copy number alterations for chromosomes 1, 3 and 8, using the Xena UCSC platform (https://xenabrowser.net/datapages/, accessed on 2 November 2023). To estimate overall survival (OS), we used survival data reported by Liu et al. [12] and employed a Kaplan–Meier estimator generated using R software (v. 4.3.1), with the survfit function from the survival library (v. 3.5-7) as well as the ggsurvplot function from the survminer library (v. 0.4.9).

## 3. Results

We employed the UMpanel to analyze a cohort of 44 patients with UM and compared the results with those generated from the TCGA-UM dataset. Our population showed an equal distribution between females (47.7%) and males (52.3%) with a median age of 62 years (range: 31–77). Tumor size ranged from 2 to 30 mm, and the predominant tumor site was the choroid (97.7%). The clinical characteristics of our population are reported in Table 1.

### 3.1. UMpanel Coverage Analysis

**Figure 1:**
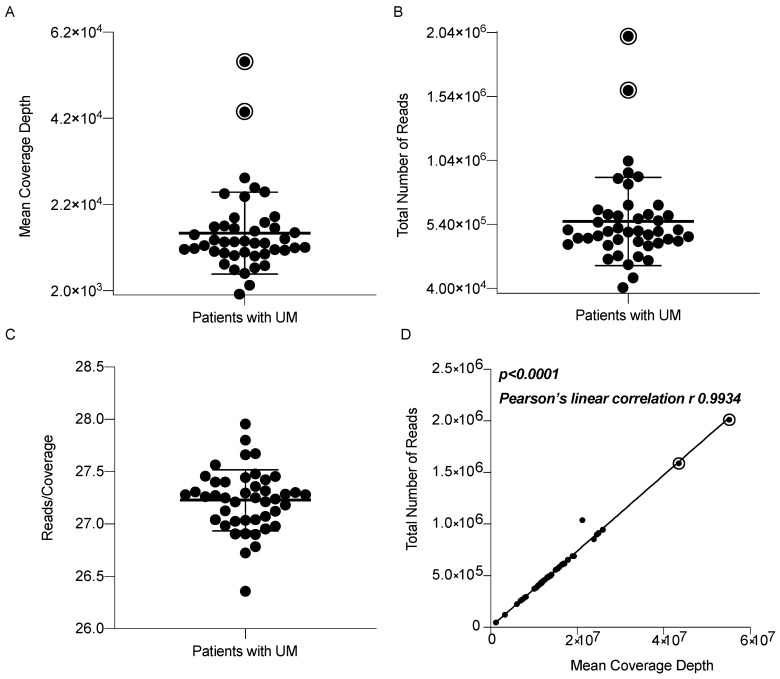
Sequencing efficiency of the UMpanel in a 44-patient cohort. (**A**–**C**) Boxplots showing the mean coverage depth (**A**), total number of reads (**B**) and reads/coverage ratio for all 44 patients (**C**). (**D**) Linear regression obtained by plotting the total number of reads vs. the mean coverage depth from each patient. Closed black dots indicate each individual patient with UM, while black dots with an open circle indicate the outliers identified using the ROUT method with the setting Q = 1% (GraphPad Prism v8).

To detect mutations and chromosomal imbalances (on chromosomes 1, 3 and 8), we used a total of 418 amplicons to sequence specific target regions (*BRAF*, *CYSLTR2*, *EIF1AX*, *GNA11*, *GNAQ*, *PLCB4* and *SF3B1*) the entire CDS (*BAP1*, *CDKN2A*, *CENPE*, *FOXO1*, *HIF1A*, *RPL5*, *SRSF2* and *TP53*) or single-nucleotide polymorphisms. Since coverage describes the number of reads encompassing a known genomic region, we wanted to establish the sequencing efficiency of our UMpanel in the entire patient cohort used in this study (Figure 1). To this end, we initially calculated the mean coverage depth, finding an average of 15.386 reads in the target regions. We identified only two outliers (95.5% overlap) which showed an even higher number of reads, suggesting a lack of sequencing gaps in the target regions (Figure 1A). We observed similar data when we plotted the total number of reads and samples with a minimum total read number of 40,000 (*n* = 44) [9]. We detected a mean of 563,630 reads (*n* = 44), with only two outliers above average, again suggesting the absence of gaps in our target regions (Figure 1B). Next, we evaluated the constant ratio between the total number of reads and the mean coverage depth for each sample. Dividing the total number of reads by the mean coverage depth, we observed a constant relationship (Figure 1C) which, when plotted as a linear regression, showed a Pearson’s r value of 0.9934 (Figure 1D). Finally, we analyzed “on-target” and “uniformity” quality metrics and found values of 96.8% for on-target regions (in silico expected value of 97.8% obtained using the AmpliSeq Designer) and 97% for uniformity coverage.

### 3.2. UMpanel Performance Validation and Comparative Analysis for Major Driver Genes

**Figure 2:**
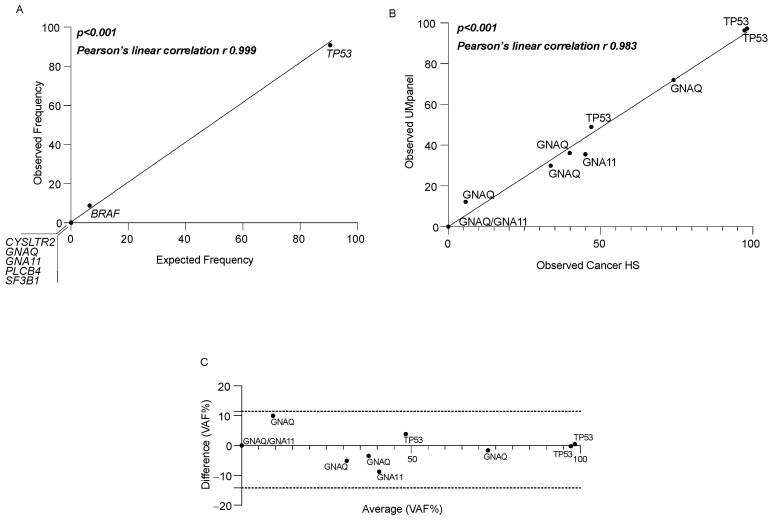
Performance evaluation of the UMpanel panel for SNV calling. (**A**) Linear regression analysis for the expected VAF vs. the observed frequency in the OncoSpan DNA reference using the UMpanel for *BRAF*, *CYSLTR2*, *GNA11*, *GNAQ*, *PLCB4*, *SF3B1* and *TP53*. VAF is expressed as a percentage derived from the average of two sequencing experiments. (**B**) Linear VAF correlation for *GNA11*, *GNAQ* and *TP53* in a comparative analysis between the UMpanel and CHPv2. VAF is expressed as a percentage derived from the average allele frequency obtained by the two gene panels. (**C**) Concordance of quantitative VAF% obtained using both the UMpanel and CHPv2 calculated as the difference vs. the average using a Bland–Altman analysis (Prism Software v.8.0). Dashed lines indicate the 95% CI as a limit of agreement between the data obtained with the two methods.

To evaluate the performance of the UMpanel, we initially used the OncoSpan DNA reference standard, which contains specific regions for *BRAF* and *TP53* that assess VAF performance. Moreover, as OncoSpan lacks the target regions for *CYSLTR2*, *GNA11*, *GNAQ*, *PLCB4* and *SF3B1*, this enabled us to evaluate the potential false positive rate. Sequencing was performed twice, and the VAF average (VAF%) for each gene was calculated by linear regression, plotting the observed vs. the expected frequency rate in our samples and obtaining a *p* < 0.001 and a Pearson’s r correlation of 0.999 (Figure 2A). To establish the ability of the UMpanel to identify different VAF values for *GNA11*, *GNAQ* and *TP53*, we applied a linear regression analysis comparing the VAFs obtained using either the UMpanel or the commercial Cancer HotSpot v2 (CHPv2). We selected eight patients with VAFs between 0 and 20%, between 30 and 60% and >60%. The patient with a 0% VAF for both *GNA11* and *GNAQ* confirmed that our UMpanel did not generate false positives and that mutations with low VAFs were not lost. Indeed, we observed a high concordance for all patients, with a *p* > 0.001 and a Pearson’s r correlation of 0.983 (Figure 2B). Lastly, we compared the VAF generated by the UMpanel with that obtained using CHPv2. Employing a Bland–Altman analysis, we plotted the difference (VAF%) vs. the average (VAF%) and found that our data were distributed between the CI 95% limits of −14.18 and +11.46 (Figure 2C). For *CDKN2A*, which is also present in CHPv2, we could not perform any comparison, as the unique mutation observed in one patient was not covered by the CHPv2 amplicons.

Lastly, we wanted to estimate the VAF concordance between the two methods for *GNA11*, *GNAQ* and *TP53.* We found high concordance between the UMpanel and CHPv2 in terms of sensitivity, specificity and Cohen’s k value (Table 2).

### 3.3. Validation of UMpanel SNV Calling by a Comparative Analysis with Conventional Sanger Sequencing and with the Genome in a Bottle Consortium Data

To validate the variant calling for most UM driver genes including *BAP1*, *CYSLTR2*, *EIF1AX*, *GNA11*, *GNAQ*, *PLCB4* and *SF3B1* (we also included the unique patient displaying a *BRAF* mutation), we performed Sanger sequencing on specific mutated target regions identified by the UMpanel (Supplementary Figure S1). We observed a 91% concordance overlap (10/11). In our analysis, the p.S154N (c.461 G>A) mutation in *CYSLTR2* was the only outlier, as this substitution was detected by NGS but not by Sanger sequencing.

The Genome in a Bottle (GIAB) consortium (https://www.nist.gov/programs-projects/genome-bottle, accessed on 25 November 2024) is a public–private academic consortium hosted by the National Institute of Standard Technology (NIST) that has characterized the pilot genome known as NA12878 from the HapMap project. To establish whether the UMpanel would identify the reported nucleotide variants, we sequenced the NA12878 genome according to the target regions present in the UMpanel. Of the 104 nucleotide variants called, only 1 rs (chr1:189235262, rs10921611, genotype C>T) generated controversial data. Indeed, although the UMpanel coverage for this target region was >500×, it failed to identify the rs10921611 SNP as reported in the IGV analysis (Supplemental Figure S2).

### 3.4. Validation of Bi-Allelic Imbalances Detected by the UMpanel

**Figure 3:**
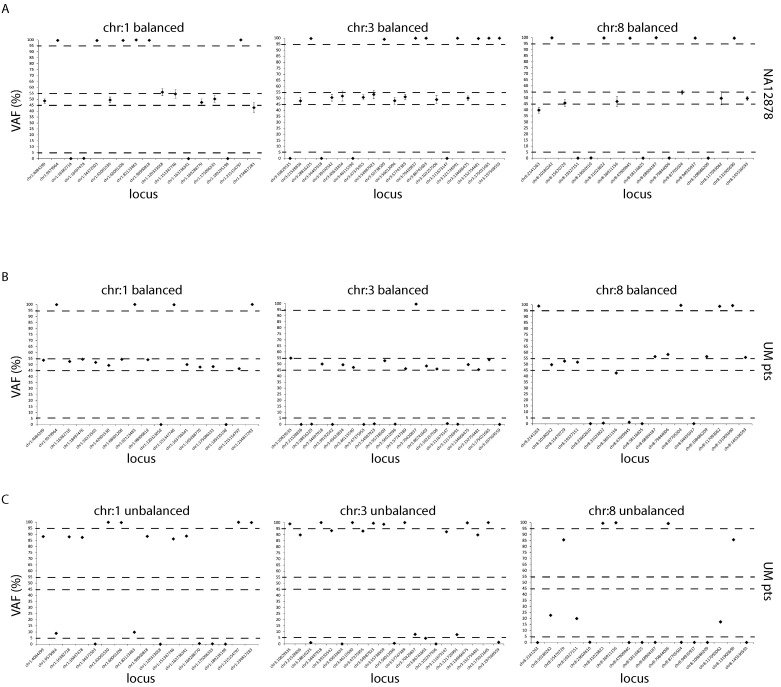
Performance evaluation of the UMpanel for the detection of imbalances in chromosomes 1, 3 and 8. (**A**,**C**) Dot plots showing each investigated SNP expressed as a VAF percentage using an arbitrary deviation of 0–5% and 95–100% for the homozygosis range and +/−5% (from 45% to 55%) for the heterozygosis range of the bi-allelic frequency. Chromosomal imbalance for NA12878 (**A**) or for patients with UM (**B**,**C**) was called when each VAF was outside of these thresholds. For all panels, the x axis shows the chromosome location for each SNP, while the y axis displays the VAF percentage. Dashed lines indicate the heterozygosis and homozygosis range, respectively.

To determine whether the UMpanel allows the reliable detection of allelic imbalances in chromosomes 1, 3 and 8, we used SNP genotyping measured by an analysis of highly polymorphic single nucleotides amplifying specific genomic regions for each chromosome arm (Supplementary Table S2). In designing the UMpanel, we included amplicons that identify SNPs located in both arms of chromosomes 1, 3 and 8, and could therefore evaluate the bi-allelic imbalance for each of them. To this end, we again employed the NA12878 HapMap sample displaying a normal bi-allelic distribution and compared the ensuing data with those obtained from patients with UM (Figure 3). Amplicons and respective rs numbers are reported in Supplementary Figure S3.

As expected, DNA reference NA12878 showed a balanced distribution for every single nucleotide, with a VAF around 50% for SNPs in heterozygosis and a VAF near 0% or 100% for those in homozygosis (Figure 3A). When we repeated the same analysis for patients with UM, we observed a normal SNP distribution, indicating the absence of allelic imbalances (Figure 3B), or a shifting of the SNPs to outside of the threshold, which demonstrated the presence of chromosomal imbalance (Figure 3C).

### 3.5. Molecular Landscape of 44 Patients with Uveal Melanoma Using the UMpanel

The UMpanel was designed to identify two sets of gene mutations and imbalances on chromosomes 1, 3 and 8. The first mutation set encompasses the eight UM driver genes according to the TCGA study, divided into “uveal melanoma initiating mutations” (*CYSLTR2*, *GNA11*, *GNAQ* and *PLCB4*) and “second hit mutations with prognostic impact” (*BAP1*, *EIF1AX*, *SF3B1* and *SRSF2*). The second mutation set contains seven further genes, namely, *BRAF*, *CDKN2A*, *CENPE*, *FOXO1*, *HIF1A*, *RPL5* and *TP53*. Among these genes, *BRAF* was used as a positive control due the absence of reported mutations in UM, while the remaining genes were included as emerging biomarkers for disease prognosis [13,14,15]. Chromosomal imbalance was studied with SNP genotyping employing single-nucleotide polymorphisms located on both arms of chromosomes 1, 3 and 8.

**Figure 4:**
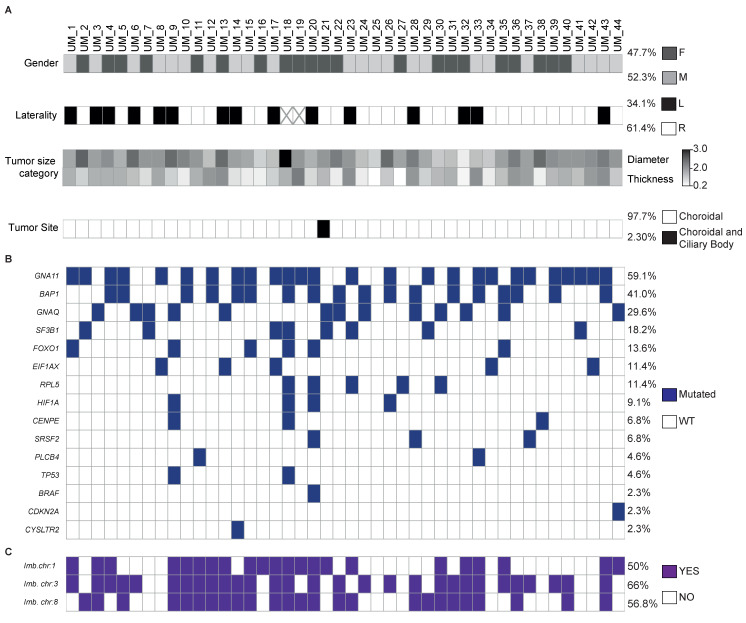
Molecular landscape of 44 patients with UM determined using the UMpanel. (**A**) Tile plots reporting percentages for gender distribution, laterality (x indicates undetermined sample; L: left eye; R: right eye), tumor size expressed in millimeters and tumor site for each patient. (**B**,**C**) Tile plots displaying mutated genes (**B**) and chromosomal imbalance (**C**).

We determined the molecular landscape of 44 patients with UM, equally distributed between females (47.7%) and males (52.3%) with a laterality of 34.1% for the left eye and 61.4% for the right eye. Tumor size was between 2 and 30 mm and, as expected, the predominant tumor site was the choroid (97.7%, *n* = 43) [16], with only one patient (2.3%, *n* = 1) presenting both a choroidal and a ciliary body tumor (Figure 4A). Our mutational analysis revealed that *GNAQ*, *GNA11* and *BAP1* were the most frequently mutated genes. For *GNAQ* and *GNA11*, we found mutation rates of 59.1% (*n* = 26) and 29.6% (*n* = 13), respectively (88.7% overall), which were mutually exclusive. For *BAP1*, the mutation rate was 41% (*n* = 18), in agreement with previously published findings [17,18] (Supplementary Table S5). A lower mutation percentage was observed for the remaining genes including *SF3B1* (18.2%, *n* = 8), *FOXO1* (13.6%, *n* = 6), *RPL5* and *EIF1AX* (11.4%, *n* = 5), *HIF1A* (9.1%, *n* = 4), *CENPE* and *SRSF2* (6.8%, *n* = 3), *PLCB4* and *TP53* (4.6%, *n* = 2), *BRAF* (non-V600E) and *CDKN2A* and *CYSLTR2* (2.3%, *n* = 1) (Figure 4B). All mutations are reported in Supplementary Table S6. Lastly, chromosomal imbalance was detected at a rate of 50% (*n* = 22) for chromosome 1, 66% (*n* = 29) for chromosome 3 and 56.8% (*n* = 25) for chromosome 8 (Figure 4C).

### 3.6. Impact of BAP1 Mutations and Bi-Allelic Imbalances in Chromosomes 1, 3 and 8 on the Survival of Patients with UM

**Figure 5:**
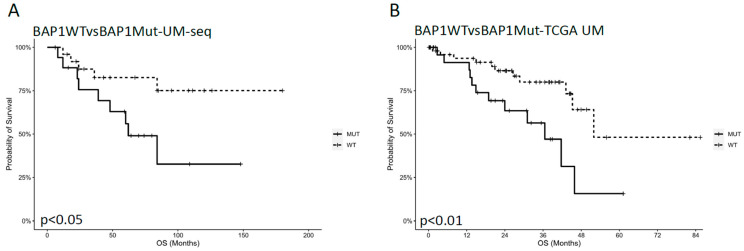
Kaplan–Meier estimator showing the impact of *BAP1* mutations on the OS of patients with UM. (**A**,**B**) Survival probability in 44 patients with UM (overall population) analyzed with the UMpanel (**A**) or in the TCGA-UM study (**B**). The x axis indicates time expressed in months, while the y axis reports OS percentages.

To establish if the UMpanel could estimate the impact of driver alterations on UM survival, we compared our OS findings with those observed in the TCGA-UM population. A *BAP1* mutation was confirmed as a negative prognostic factor [19], since in our patient cohort, *BAP1*-mutated individuals (*n* = 17; 38.63%) displayed shorter survival compared with *BAP1* WT patients (Figure 5A), as previously reported in the TCGA-UM population (Figure 5B).

**Figure 6:**
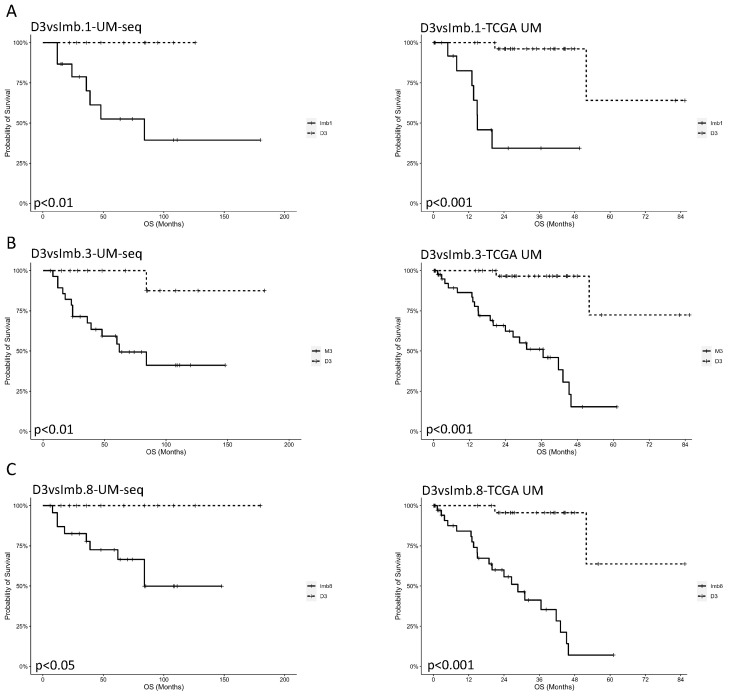
Kaplan–Meier estimator showing the impact of bi-allelic imbalances in chromosomes 1, 3 and 8 on the OS of patients with UM. (**A**–**C**) Survival probability analyzed for the UMpanel cohort (left panels) or in the TCGA-UM study (right panels), comparing patients showing D3vsImb1 (D3 *n* = 13, Imb1 *n* = 15) (**A**), D3vsImb3 (D3 *n* = 15, Imb3 *n* = 29) (**B**) or D3vsImb8 (D3 *n* = 11, Imb8 *n* = 24) (**C**). The x axis indicates time expressed in months, while the y axis reports OS percentages. D3 = chromosome 3 disomy; Imb 1 = imbalance in chromosome 1; Imb 3 = imbalance in chromosome 3; Imb 8 = imbalance in chromosome 8.

As for chromosomal imbalances, the TCGA-UM study segregated patients into two prognostic groups based on the presence or absence of chromosome 3 monosomy alone or associated with chromosome 1 and 8 aberrations. We compared our cohort with the TCGA-UM dataset using the disomy of chromosome 3 as a baseline to calculate the OS of five different groups displaying an imbalance in chromosomes 3, 1 and 8 alone or combined (Figure 6 and Supplementary Figure S4). In our UM cohort, all patients with bi-allelic imbalances in chromosomes 1, 3 or 8 showed inferior OS when compared with those presenting a disomy of chromosome 3 (Figure 6A–C, left panels). We observed similar data when we analyzed the OS of patients displaying the combination of imbalances in chromosome 3 associated with chromosome 1 or chromosome 8 alterations (Supplementary Figure S4A,B, left panels). For both analyses, our findings were comparable with those reported in the TCGA-UM study (Figure 6A–C, right panels) (Supplementary Figure S4A,B, right panels).

## 4. Discussion

The concept of precision medicine postulates that individuals diagnosed with the same disease may present distinct genetic alterations, influencing therapeutic responses and clinical outcomes [19]. Indeed, the identification of such genetic differences has already improved the diagnostic and therapeutic approaches employed for several tumor types [20,21,22,23]. Uveal melanoma is a deadly disease caused by a specific set of mutated genes and chromosomal aberrations, associated with the risk of developing distant metastases [24]. Several prognostic biomarkers have been investigated in UM, including *BAP1* mutations and chromosomal abnormalities [25]. However, a standardized approach to define the genetic profile of patients with UM has not yet been defined.

Expanding the results of the TCGA-UM project, we provide a custom DNA-based NGS panel—named the UMpanel—associated with an optimized pipeline capable of generating a genetic profile for patients with UM that reliably estimates their survival probability after surgical intervention. Our workflow is cost-effective, simple, accurate and can replace multiple laborious techniques such as FISH, SNP arrays and Sanger sequencing. Our workflow can also detect mutations and chromosomal imbalances in one step and analyze additional biomarkers such as *CDKN2A*, *CENPE*, *FOXO1*, *HIF1A*, *RPL5* and *TP53*. These genes have recently been reported to be involved in UM pathogenesis and progression and may provide new insights into disease pathogenesis and molecular evolution [13,14,26].

When we investigated the performance of our UMpanel, we observed excellent coverage and high concordance between the different methods used for comparative analysis. Indeed, coverage analysis showed a satisfactory linear correlation, with only two outliers showing high coverage, suggesting no sequencing gaps in the target regions. When we analyzed on-target and uniformity coverage, we found comparable results with those expected by the in silico prediction analysis generated by the AmpliSeq Designer, suggesting that in the UMpanel, individual reads were distributed across each covered region of interest.

Additionally, a performance evaluation comparing our results with those generated with a standard DNA reference (OncoSpan) or with CHPv2 (the commercial Cancer HotSpot Panel) displayed 100% specificity and sensitivity, as confirmed by a Blant–Altman analysis. When we performed a comparative analysis using the Sanger method, we detected a 91% overlap. The same high-quality data were observed for imbalances in chromosomes 1, 3 and 8, as the chromosomal aberrations identified in our UM cohort were not identified in the NA12878 DNA reference. Interestingly, when we performed a comparative SNP analysis employing the NA12878 pilot genome, we observed an overlap of 99.03% between the UMpanel and GIAB sequencing, again confirming the accuracy of our workflow.

As expected, in our UM cohort, mutations in *GNA11* and *GNAQ* were highly prevalent and mutually exclusive, followed by sequence alterations in *BAP1* and *SF3B1*. Lower mutational frequencies were detected for *CYSLTR2*, *EIF1AX*, *PLCB4* and *SRSF2* [8,27]. The molecular classification of our patient cohort generated by the UMpanel allowed individual stratification based on the presence or absence of *BAP1* mutations or chromosomal abnormalities, generating OS estimates comparable to those reported by the TCGA-UM study, further validating our methodological approach.

While our NGS-based workflow displayed excellent performance levels, several issues need to be addressed. The first issue concerns the copy number variation (CNV) analysis for *BAP1* and *CDKN2A*. Both CNVs typically occur due to gene deletion, and their detection has important implications for the prognosis of patients with UM [26,28]. While the imbalance in chromosome 1 could be used as a surrogate for *BAP1* CNVs, this approach would not detect a *CDKN2A* deletion. Hence, alternative methodological approaches (e.g., fluorescence in situ hybridization for *CDKN2A*) may be required to tackle this problem. However, further analyses are in progress to establish whether the UMpanel can be used to detect *BAP1* and *CDKN2A* CNVs. A second issue concerns the limit of detection (LoD) in samples with low tumor content. In our study, all specimens displayed a tumor cellularity of 85–95%, and additional analyses in a larger patient cohorts will be needed to define the accuracy of our workflow in samples with lower tumor content. Lastly, the UMpanel does not include genes involved in the PI3K/AKT/mTOR pathway that may also display genetic alterations in patients with UM, although no correlation with individual risk categorization has yet been recognized [13,29].

## 5. Conclusions

In summary, similarly to what we previously reported for patients with glioma [30], we describe a DNA-based NGS panel suitable for the molecular classification of patients with UM who can be categorized into high- or low-risk groups. This early survival prediction may be clinically relevant, as patients with UM may receive a tailored clinical approach based on their individual risk. Furthermore, this genetic test is applicable to all patients with UM eligible for primary or secondary enucleation who may be stratified into a specific risk category, improving their clinical management. Finally, since the availability of tumor samples may represent a limit in genetic testing for patients with UM undergoing eye-preserving irradiation [31], the UMpanel has also been implemented to test cell-free DNA, and a liquid biopsy program is currently under investigation.

## Figures and Tables

**Table 1:** Clinical characteristics of patients with uveal melanoma.

Characteristic	Value
Overall population	44 (100%)
**Gender**	
Male	23 (52.3%)
Female	21 (47.7%)
**Age/years**	62 (median with range of 31–77)
**Side**	2 N.A.
Left eye	15 (35.7%)
Right eye	27 (64.3%)
**Thickness**	
≤10 (mm)	22 (50%)
>10 (mm)	22 (50%)
**Diameter**	
≤10 (mm)	10 (22.7%)
>10 (mm)	34 (77.3%)
**Tumor type**	
Choroid	43 (97.7%)
Choroid and ciliary body	1 (2.3%)
**Extraocular extension**	
Yes	3 (6.8%)
No	41 (93.2%)

N.A.: not available.

**Table 2:** Concordance between the UMpanel and CHPv2 for *GNA11*, *GNAQ* and *TP53.*

Variable	*GNA11*/*GNAQ*/*TP53*
Samples analyzed with both CHPv2 and the UMpanel	9
Positive in both the UMpanel and CHPv2	8
Positive in CHPv2 and Negative in the UMpanel	0
Positive in the UMpanel and Negative in CHPv2	0
Negative in both the UMpanel and CHPv2	1
Cohen’s k	1
Sensitivity for CHPv2, %	100
Specificity for CHPv2, %	100
Sensitivity for NGS, %	100
Specificity for NGS, %	100

## Data Availability

The original contributions presented in the study are included in the article, further inquiries can be directed to the corresponding author.

## Supplementary Materials

The following supporting information can be downloaded at https://www.mdpi.com/article/10.3390/biom15010146/s1, Figure S1: Comparative analysis between the Sanger pherogram and IGV report of the NGS analysis for each indicated mutation; Figure S2: IGV-derived images of rs1092161; Figure S3: IGV-derived images showing the location of each SNP used for chromosome imbalance analysis; Figure S4: OS of patients with imbalances in CHR:3 + 1 and CHR:3 + 8; Table S1: Genomic coordinates of the amplicon for the 15-gene panel; Tables S2 and S3: List of the SNPs covered by the uveal melanoma panel; Table S4: Primer sequence for each gene analyzed with the Sanger method; Table S5: Comparative analysis of VAF% for *GNAQ*, *GNA11* and *BAP1* genes with published data; Table S6: Clinical variants (SNVs, Ins/Dels).
