# Supplementary material for: An Optimized NGS Workflow Defines Genetically Based Prognostic Categories for Patients with Uveal Melanoma
Source: Biomolecules. 2025 Jan 18;15(1):146. doi: 10.3390/biom15010146 (PMC11763870; doi:10.3390/biom15010146)
Supplement: Supplementary file 1 [file biomolecules-15-00146-s001.zip › Figure S1.pdf]

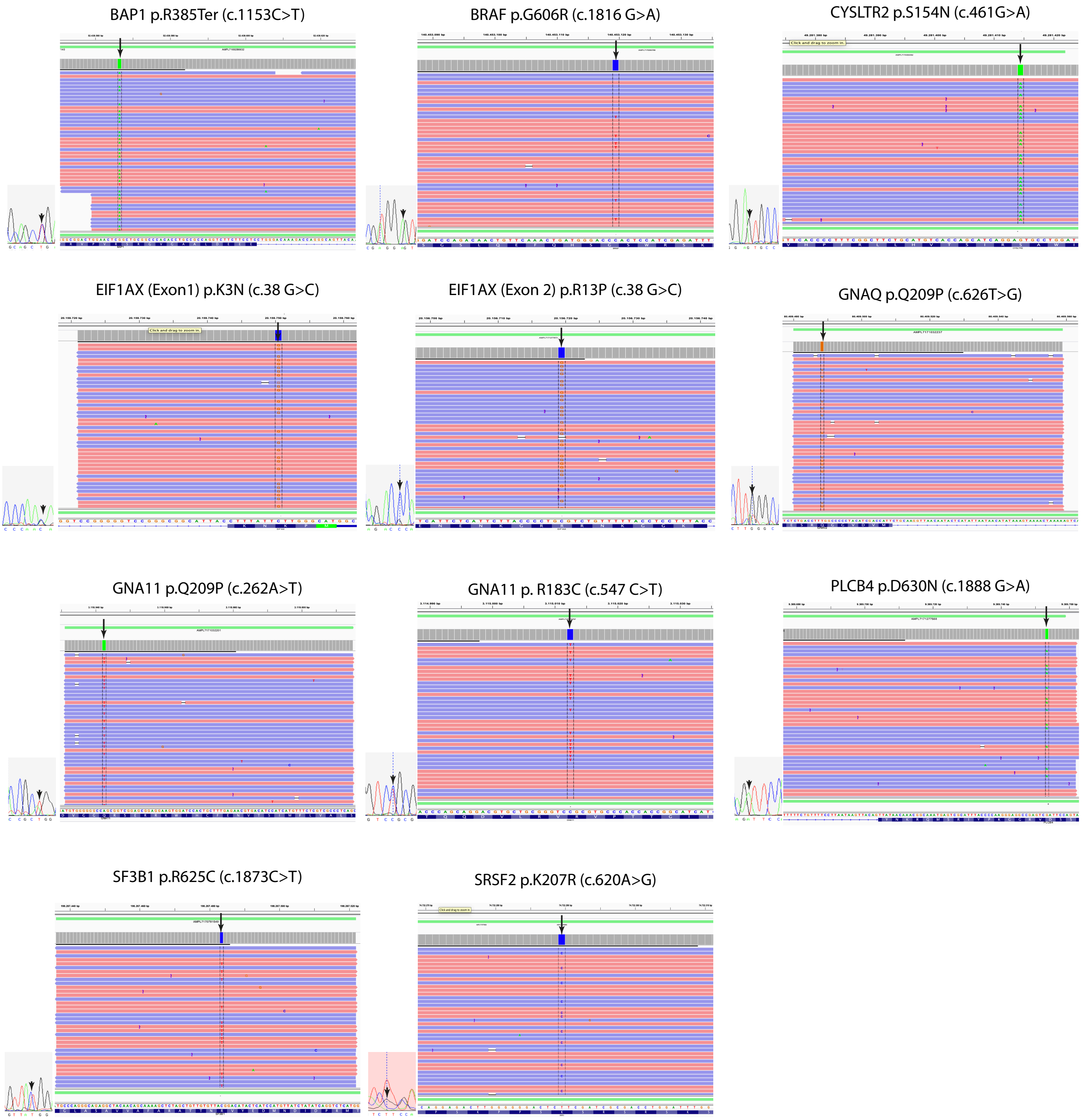

Figure S1. Comparative analysis between the UMpanel and conventional Sanger sequencing. Each image, derived from the Integrative Genomic Viewer (IGV), reports the forward (pink) and reverse (violet) reads for each amplico sequenced by NGS. Small inserts in the lower left corner of each image indicate the pherograms generated by Sange sequencing. Black arrows indicate the SNV calling for both IGV and Sanger, with p. referring to the amino acidic substitution and c. denoting the nucleotide substitution in the coding region. For all panels, each arrow indicates the nucleotide substitution according to A=green, C=blu and G=orange.
