# Supplementary material for: An Optimized NGS Workflow Defines Genetically Based Prognostic Categories for Patients with Uveal Melanoma
Source: Biomolecules. 2025 Jan 18;15(1):146. doi: 10.3390/biom15010146 (PMC11763870; doi:10.3390/biom15010146)
Supplement: Supplementary file 1 [file biomolecules-15-00146-s001.zip › Figure S2.pdf]

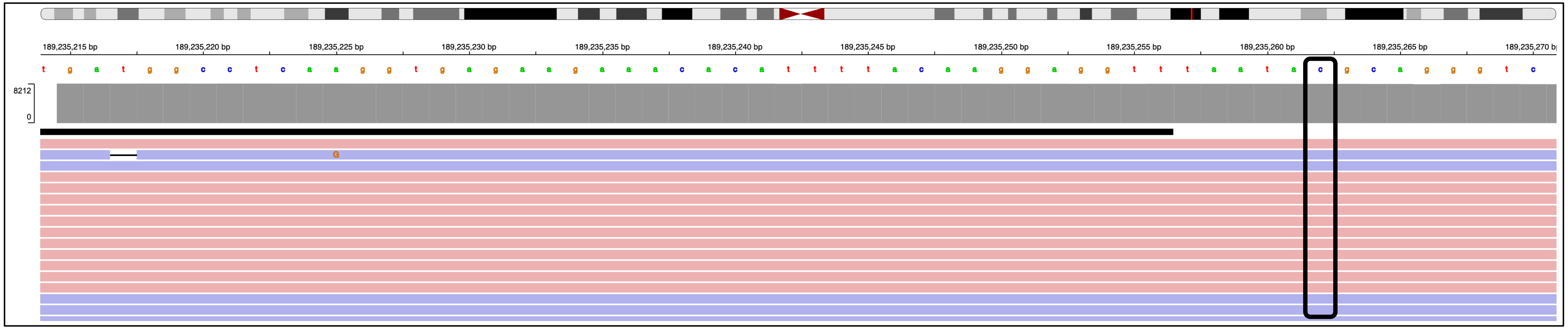

Figure S2: Picture showing the alignment by IGV for expected SNP according to the GIAB sequencing. The black square indicates the chr1:189235262, rs10921611, genotype C>T detected by GIAB but not identified by UMpanel although the high coverage observed for this target region.
