# Supplementary material for: An Optimized NGS Workflow Defines Genetically Based Prognostic Categories for Patients with Uveal Melanoma
Source: Biomolecules. 2025 Jan 18;15(1):146. doi: 10.3390/biom15010146 (PMC11763870; doi:10.3390/biom15010146)
Supplement: Supplementary file 1 [file biomolecules-15-00146-s001.zip › Figure S4.pdf]

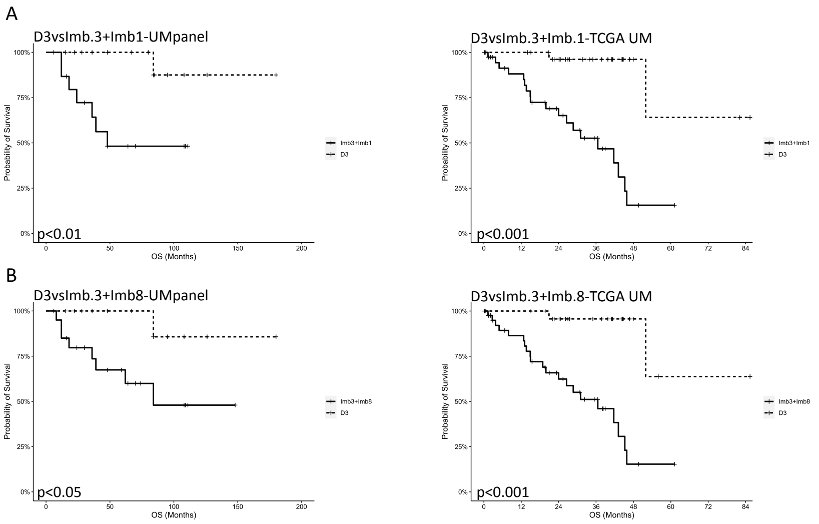

Figure S4. Kaplan-Mayer estimator showing the impact of Bi-allelic imbalances of chromosomes 1, 3 and 8 on OS of UM patients. **A** and **B**: Survival probability obtained from UM analyzed by UMpanel (left panels) or TCGA-UM study (right panel) obtained from patients diasplaying D3vsImb3+Imb1 (D3 n=16, Imb3+Imb1 n=16) (**A**), or D3vsImb3+Imb8 (D3 n=14, Imb3+Imb8 n=21) (**B**). X axis indicates the time expressed in months, while y axis reports OS expressed as percentage.
