# Supplementary material for: An Optimized NGS Workflow Defines Genetically Based Prognostic Categories for Patients with Uveal Melanoma
Source: Biomolecules. 2025 Jan 18;15(1):146. doi: 10.3390/biom15010146 (PMC11763870; doi:10.3390/biom15010146)
Supplement: Supplementary file 1 [file biomolecules-15-00146-s001.zip › Table S1.pdf]

**Table S1**. Genomic coordinates of the amplicons used for the analysis of the 15 genes included in the UMpanel (reference sequence: hg19/grch37)

| Gene | Genomic coordinates (start - stop) | Sequenced Regions |
|---|---|---|
| *BAP1* | chr3: 52436229-52443912 | All coding exons±10bp |
| *CDKN2A* | chr9: 21968185-21994373 | All coding exons±10bp |
| *CENPE* | chr4: 104027367-104119529 | All coding exons±10bp |
| *FOXO1* | chr13: 41133599-41240372 | All coding exons±10bp |
| *HIF1A* | chr14: 62162449-62213826 | All coding exons±10bp |
| *RPL5* | chr1: 93297641-93307456 | All coding exons±10bp |
| *SRSF2* | chr17: 74732194-74733315 | All coding exons±10bp |
| *TP53* | chr17: 7572852-7579924 | All coding exons±10bp |
| *BRAF* | chr7: 140453086-140453170 | Exon 15 |
| *CYSLTR2* | chr13: 49281338-49281421 | Exon 1 |
| *EIF1AX* | chrX: 20159723-20160019 | Exon 1±10bp |
| *EIF1AX* | chrX: 20156571-20156757 | Exon 2±10bp |
| *GNA11* | chr19: 3118930-3119014 | Exon 5 |
| *GNA11* | chr19: 3114958-3115053 | Exon 4 |
| *GNAQ* | chr9: 80409479-80409558 | Exon 5 |
| *GNAQ* | chr9: 80412414-80412506 | Exon 4 |
| *PLCB4* | chr20: 9389675-9389758 | Exon 20 |
| *SF3B1* | chr2: 198267435-198267521 | Exon 14 |
