# Supplementary material for: An Optimized NGS Workflow Defines Genetically Based Prognostic Categories for Patients with Uveal Melanoma
Source: Biomolecules. 2025 Jan 18;15(1):146. doi: 10.3390/biom15010146 (PMC11763870; doi:10.3390/biom15010146)
Supplement: Supplementary file 1 [file biomolecules-15-00146-s001.zip › Table S2.pdf]

Table S2. List of the highly polymorphic single nucleotide polymorphisms (SNP) covered by the uveal melanoma panel

	SNP-number	Position (bp)
Chromosome 1	rs7418256	4,084,304
	rs7412149	9,579,964
	rs12048851	16,382,718
	rs10907287	18,497,478
	rs6425861	34,372,503
	rs639298	42,001,530
	rs11209106	68,001,206
	rs480304	82,123,485
	rs10493903	98,900,818
	rs17258467	120,323,058
	rs1752380	151,347,746
	rs3856201	163,736,341
	rs10753786	169,288,770
	rs2072040	175,096,333
	rs12144406	189,235,198
	rs6681013	215,154,797
	rs592197	234,817,283
Chromosome 3	rs1601368	10,829,535
	rs1549356	21,528,837
	rs7612272	28,816,226
	rs7648156	34,497,918
	rs1274960	39,192,542
	rs267218	45,633,834
	rs9311387	46,115,590
	rs295449	47,375,955
	rs3821659	54,987,923
	rs2702143	55,738,509
	rs9868630	56,012,096
	rs62259027	57,747,389
	rs9310190	70,420,837
	rs12497448	86,741,603
	rs11511334	102,257,506

	rs3749299	111,673,147
	rs1523517	121,726,991
	rs975149	134,666,475
	rs1004009	152,754,481
	rs9866779	175,021,665
	rs11717776	197,569,559
Chromosome 8	rs2405488	2,141,263
	rs4498602	10,180,242
	rs17577614	15,470,726
	rs13275706	19,327,151
	rs6557699	23,602,610
	rs1882928	31,023,822
	rs10095600	36,911,156
	rs4147426	47,909,945
	rs76405556	58,116,825
	rs6995640	68,904,187
	rs2120410	79,844,006
	rs13261311	87,705,504
	rs4735258	94,935,937
	rs4734993	108,686,209
	rs2142250	117,093,062
	rs6415522	131,905,690
	rs7008457	145,536,593
