# Supplementary material for: An Optimized NGS Workflow Defines Genetically Based Prognostic Categories for Patients with Uveal Melanoma
Source: Biomolecules. 2025 Jan 18;15(1):146. doi: 10.3390/biom15010146 (PMC11763870; doi:10.3390/biom15010146)
Supplement: Supplementary file 1 [file biomolecules-15-00146-s001.zip › Table S3.pdf]

Table S3. Genomic coordinates, of the amplicons used for the analysis of the 55 SNPs included in the UMpanel (reference sequence: hg19/grch37)

Chromosome	START	END	ID_rs	LOCATION
chr1	4084266	4084353	GENE_ID=1p_rs7418256;	None
chr1	9579943	9580034	GENE_ID=1p_rs12048851;	None
chr1	16382708	16382793	GENE_ID=1p_rs12048851;	CLCNKB:intronic:NM_000085.5
chr1	18497465	18497547	GENE_ID=1p_rs10907287;	IGSF21:intronic:NM_032880.5
chr1	34372436	34372524	GENE_ID=1p_rs6425862;	CSMD2:intronic:NM_052896.4
chr1	42001468	42001559	GENE_ID=1p_rs639298;	HIVEP3:intronic:NM_001127714.3
chr1	68001121	68001210	GENE_ID=1p_rs11209106;	None
chr1	82123447	82123527	GENE_ID=1p_rs480304;	None
chr1	98900763	98900849	GENE_ID=1p_rs10493903;	None
chr1	120323040	120323133	GENE_ID=1p_rs17258467;	None
chr1	151347724	151347820	GENE_ID=1q_rs1752380;	None
chr1	163736312	163736399	GENE_ID=1q_rs3856201;	None
chr1	169288727	169288812	GENE_ID=1q_rs10753786;	NME7:intronic:NM_013330.5
chr1	175096302	175096390	GENE_ID=1q_rs2072040;	TNN:intronic:NM_022093.2
chr1	189235181	189235270	GENE_ID=1q_rs12144406;	None
chr1	215154760	215154849	GENE_ID=1q_rs6681013;	None
chr1	234817241	234817329	GENE_ID=1q_rs592197;	None
chr3	10829486	10829567	GENE_ID=3_rs1601368;	None
chr3	21528775	21528861	GENE_ID=3_rs1549356;	ZNF385D:intronic:NM_024697.3
chr3	28816171	28816256	GENE_ID=3_rs7612272;	None
chr3	34497903	34497987	GENE_ID=3_rs7648156;	None
chr3	39192460	39192553	GENE_ID=3_rs1274960;	CSRNP1:intronic:NM_033027.4
chr3	45633786	45633872	GENE_ID=3_rs267218;	None
chr3	46115512	46115596	GENE_ID=3_rs9311387;	None
chr3	47375927	47376016	GENE_ID=3_rs295449;	KLHL18:intronic:NM_025010.5
chr3	54987920	54988011	GENE_ID=3_rs3821659;	CACNA2D3:intronic:NM_018398.3
chr3	55738450	55738532	GENE_ID=3_rs2702143;	ERC2:intronic:NM_015576.3
chr3	56012091	56012179	GENE_ID=3_rs9868630;	ERC2:intronic:NM_015576.3
chr3	57747342	57747427	GENE_ID=3_rs62259027;	SLMAP:intronic:NM_007159.5
chr3	70420799	70420879	GENE_ID=3_rs9310190;	None
chr3	86741547	86741636	GENE_ID=3_rs12497448;	None
chr3	102257455	102257542	GENE_ID=3_rs1151334;	None
chr3	111673118	111673189	GENE_ID=3_rs3749299;	PHLDB2:intronic:NM_001134439.2
chr3	121726977	121727041	GENE_ID=3_rs1523517;	ILDR1:intronic:NM_001199799.2
chr3	134666440	134666525	GENE_ID=3_rs975149;	EPHB1:intronic:NM_004441.5
chr3	152754435	152754513	GENE_ID=3_rs1004009;	None
chr3	175021618	175021707	GENE_ID=3_rs9866779;	NAALADL2:intronic:NM_207015.3
chr3	197569486	197569570	GENE_ID=3_rs11717776;	LRCH3:intronic:NM_032773.3
chr8	2141201	2141285	GENE_ID=8p_rs2405488;	None
chr8	10180223	10180298	GENE_ID=8p_rs4498602;	MSRA:intronic:NM_012331.5
chr8	15470685	15470766	GENE_ID=8p_rs17577614;	TUSC3:intronic:NM_006765.4
chr8	19327063	19327156	GENE_ID=8p_rs13275706;	CSGALNACT1:intronic:NM_018371.4
chr8	23602568	23602651	GENE_ID=8p_rs6557699;	None
chr8	31023787	31023873	GENE_ID=8p_rs1882928;	WRN:intronic:NM_000553.6
chr8	36911140	36911220	GENE_ID=8p_rs10095600;	None
chr8	47909881	47909961	GENE_ID=8q_rs4147426;	None
chr8	58116820	58116908	GENE_ID=8q_rs76405556;	None

chr8	68904156	68904238	GENE_ID=8q_rs6995640;	PREX2:intronic:NM_024870.4
chr8	79843960	79844046	GENE_ID=8q_rs2120410;	None
chr8	87705443	87705524	GENE_ID=8q_rs13261311;	CNGB3:intronic:NM_019098.5
chr8	94935904	94935990	GENE_ID=8q_rs4735258;	PDP1:utr_3:NM_001161780.2
chr8	108686145	108686220	GENE_ID=8q_rs4734993;	None
chr8	117092976	117093063	GENE_ID=8q_rs2142250;	LINC00536:intronic_nc:NR_046215.1
chr8	131905645	131905734	GENE_ID=8q_rs6415522;	ADCY8:intronic:NM_001115.3
chr8	145536554	145536644	GENE_ID=8q_rs7008457;	HSF1:intronic:NM_005526.4
