# Supplementary material for: An Optimized NGS Workflow Defines Genetically Based Prognostic Categories for Patients with Uveal Melanoma
Source: Biomolecules. 2025 Jan 18;15(1):146. doi: 10.3390/biom15010146 (PMC11763870; doi:10.3390/biom15010146)
Supplement: Supplementary file 1 [file biomolecules-15-00146-s001.zip › Table S4.pdf]

Table S4. Primers sequence for each analyzed gene by Sanger method

GENE	Primer Forward	Primer Reverse	Amplicon size pb
GNAQ (exon 5)	*AGTATTGTTAACCTTGCAG*	*CGAGAACTTGATCATATTC*	*132*
GNA11 (exon 5)	*GTCCTGGGATTGCAGATT*	*GGCGACGAGAAACATGAT*	*154*
GNA11 (exon 4)	*GACCGCATCGCCACCTT*	*CAGGTCGAAAGGGTACTC*	*96*
BAP1 (exon 12)	*GAGTTGGTGTTCTGCACG*	*GTCTTTGTCCCAGGAGGA*	*138*
BRAF (exon 15)	*TGGATCCAGACAACTGT*	*ACAGTAAAAATAGGTGATTTT*	*85*
PLCB4 (exon 23)	*GTTACAGTTATAACAAACGGC*	*GATAGTTCAGTGAAACCATC*	*123*
SF3B1 (exon 14)	*CTGCTGGTCTGGCTACTAT*	*CCAGGACTTCTTGCTTTTGC*	*167*
SRSF2 (exon 2)	*TTCCTCTTCAGGAGACTTGG*	*GTCCCGGTCTCGGTCC*	*97*
CYSLTR2 (exon 1)	*CTGACCGTGCTGAGTGTT*	*CCAGGCACTCCTGATGC*	*84*
EIF1AX (exon 1)	*AATCTACGGGCAGGACCT*	*CTCCAGCACCTACTTGGT*	*200*
EIF1AX (exon 2)	*GCGTAATTTATGAAAACACTTA*	*TTTATTTCATACTGTTTTACAG*	*174*
