# Supplementary material for: An Optimized NGS Workflow Defines Genetically Based Prognostic Categories for Patients with Uveal Melanoma
Source: Biomolecules. 2025 Jan 18;15(1):146. doi: 10.3390/biom15010146 (PMC11763870; doi:10.3390/biom15010146)
Supplement: Supplementary file 1 [file biomolecules-15-00146-s001.zip › Table S5.pdf]

Table S5. Comparative analysis of VAF(%) observed in our analysis with that reported in previous published data of the indicated genes.

<table>
<tr><th colspan="4">COMPARATIVE ANALISYS OF VAF% ACCORDING TO THE PUBLISHED DATA</th></tr>
<tr><th>MUTATED GENES</th><th>VAF% OBSERVED</th><th>VAF% EXPECTED (RANGE%)</th><th>REFERENCES (doi)</th></tr>
<tr><td>GNA11</td><td>59.1%</td><td>24.2%-60%</td><td rowspan="2">10.3390/cancers14133066, 10.1038/bjc.2013.299, 10.1007/s12253-017-0371-7, 10.1136/bjo.2009.174417, 10.1111/aos.14760, 10.21873/anticanres.12010</td></tr>
<tr><td>GNAQ</td><td>29.8%</td><td>24.2%-53.3%</td></tr>
<tr><td>BAP1</td><td>41%</td><td>40%-47%</td><td>10.1038/s41598-021-88390-6, 10.1126/science.1194472, 10.1038/s41467-020-15606-0, 10.3390/cancers14174105</td></tr>
</table>
